# Association between waterpipe smoking and obesity: Population-based study in Qatar

**DOI:** 10.18332/tid/143878

**Published:** 2022-01-26

**Authors:** Abdulla A. Alkeilani, Abdelrahman A. Khalil, Afaf M. Azzan, Noof A. Al-Khal, Noora H. Al-Nabit, Omar M. Talab, Rahaf A. Al-Hajri, Samreen M. Rahmoon, Anas A. Ashour, Ishita Gupta, Ala-Eddin Al Moustafa

**Affiliations:** 1 College of Medicine, University Health, Qatar University, Doha, Qatar; 2 Department of Internal Medicine, Hamad Medical Corporation, Doha, Qatar; 3 Biomedical and Pharmaceutical Research Unit, Qatar University Health, Qatar University, Doha, Qatar; 4 Biomedical Research Centre, Qatar University, Doha, Qatar; 5 Department of Oncology, Faculty of Medicine, McGill University, Montreal, Canada

**Keywords:** waterpipe, smoking, obesity, body mass index, Qatar

## Abstract

**INTRODUCTION:**

Over the past decade obesity prevalence has been increasing rapidly in the Gulf region (GR) including Qatar, becoming one of the major health issues in the region. Concomitantly, waterpipe (WP) smoking is increasing worldwide especially in the GR, and although the effect of cigarette smoking on body weight is well-established, studies indicating an association between WP smoking and obesity are scarce. Thus, we explored the association between WP smoking and obesity in comparison with cigarette smokers and healthy population in Qatar.

**METHODS:**

We performed a cross-sectional study using data from Qatar Biobank and analyzed anthropometric measurements among 879 adults (aged 18–65 years) that included WP smokers, cigarette smokers, dual smokers and never smokers. Body composition was measured using bioelectrical impedance analysis and reported as lean mass, fat mass, and body fat percentage.

**RESULTS:**

Overall, 12% (n=108) were WP smokers, 22% (n=196) were cigarette smokers, 9% (n=77) smoked both WP and cigarettes and 57% (n=498) were never smokers. Age, sex, history of diabetes, and hypertension, in addition to nationality were considered as confounding factors. Our analysis revealed that WP smokers had a significantly higher BMI (kg/m^2^) and fat mass when compared with cigarette smokers (p<0.05). Moreover, compared to cigarette smoking, WP smoking had a higher significant effect on BMI (β=3.8, SE=0.38; and β=5.5, SE=0.46; respectively), and fat mass (β=5.1, SE=0.79; and β=9.0, SE=0.97; respectively). However, WP users were similar to never-smokers in terms of body fat percent.

**CONCLUSIONS:**

Our data indicate that compared to never smokers, daily WP users have higher BMI and fat mass, and are likely to be obese.

## INTRODUCTION

In recent years, there has been a universal gradual shift from cigarette smoking towards waterpipe (WP) smoking and e-cigarettes with almost 100 million smokers consuming WP on a daily basis^1^. The rise in WP smoking trend is mainly due to the fact that some WP users consider this practice less harmful than smoking cigarettes, since inhaling smoke flavored with aromatic fruit through hookah water is believed to be less toxic^2^. However, research shows that both methods of tobacco consumption (WP smoking and cigarette smoking) modify gene expression regulating detoxification, xenobiotic metabolism, as well as DNA stability and repair processes^3^, resulting in severe health problems that are involved in a variety of oral and systemic diseases, such as periodontal affliction, heart disease, pulmonary disorders, and multiple types of cancer^4^. Moreover, we recently reported that WP smoking can exhibit a substantial embryotoxicity in the early stage of embryogenesis^5^.

Obesity is also becoming one of the most serious health conditions worldwide including the Gulf region (GR)^6^. This disease correlates with several comorbidities leading to an increase in disability, morbidity, and mortality^6^. In Qatar, obesity is considered as a major risk factor contributing to serious health issues and has reached epidemic levels affecting 41.4% of all Qatari nationals (39.5% men and 43.2% women)^7^. The two most common factors associated with obesity in adults are high-caloric diet and lack of physical activity^8^. However, several studies have also identified smoking including WP smoking as a risk factor for obesity^9,10^. A study conducted in the UK found that the risk of obesity was higher in current smokers in a dose-dependent manner; among smokers, the risk of obesity increased with the amount smoked, however, compared to former light smokers, former heavy smokers were probably more obese^11^. In addition, the study found that after almost three decades of quitting smoking, former smokers were at the same risk of obesity as never smokers in comparison with current smokers; however, the authors failed to provide evidence for any protective effect of smoking against weight gain in sub-groups of young people and women^11^. Another investigation in Syria found that daily WP smokers have a higher body mass index (BMI)^12^. Although WP smoking could be a suspected risk factor for obesity, association of WP smoking with body weight or overall obesity is still unknown. Thus, we explored the associations of WPS with BMI and obesity status using data from the Qatar BioBank (QBB); our data indicate that WP users, compared to never smokers, have higher BMI and are likely to be obese.

## METHODS

### Study design and population

We conducted a cross-sectional study using data from the QBB project (https://www.qatarbiobank.org.qa), which is a longitudinal cohort study that was initiated in December 2012, in adults aged 18–89 years from the population in Qatar. Only Qatari adults or long-term residents of Qatar (≥15 years) were eligible to participate in the QBB platform. Participation was encouraged by social media or family and friends’ recommendations^13^.

Inclusion criteria for participation were written consent and completion of a self-administered questionnaire about demographic and social characteristics, lifestyle factors, and past medical history. A series of anthropometric measurements and blood tests were then conducted. Exclusion criteria included subjects going on special diets or weight control medications, use of cortico-steroid medications, drug or alcohol abuse, long standing comorbidities (diabetes, hypertension, chronic thyroid diseases, asthma, coronary artery disease, or cancer) of ≥15 years, females that had a recent delivery, and past smokers. Our study collected a random sample of 1000 participants aged 18–65 years to explore the association between WPS and obesity. A total of 121 subjects were excluded due to missing data, thus forming a total sample of 879 subjects who were divided based on their smoking status and included in the analysis.

### Smoking status and lifestyle

Participants who reported active WP smoking at the time of recruitment were considered as WP smokers. The majority of WP smokers reported smoking one session per day, hence, were not further classified. Cigarette smoking was calculated by the number of cigarettes smoked per day. Individuals who reported no history of WPS or cigarette smoking were considered never smokers. To avoid misclassification bias, participants who used to smoke but stopped smoking were excluded from the study. Information on physical activity was obtained by administering questions using a concise version of the International Physical Activity Questionnaire (IPAQ)^14^. However, questions with a large number of missing responses were not included in the study. Physical activity was measured by the frequency of performing various activities and reported as number of days per week. In addition, details about occupational status and degree of physical activity at work were collected.

### Anthropometric measurements

The height (m) and weight (kg) were measured with participants wearing light clothes and no shoes. The height and weight were measured once using a wall-mounted stadiometer under the supervision of a registered nurse. The instrument was calibrated after each participant. Body mass index (BMI, kg/m^2^) was calculated and categorized into four groups: underweight (<18.5), normal weight (18.5–24.9), overweight (25.0–29.9), and obese (>30). Additionally, body composition was measured using bioelectrical impedance analysis and reported as lean mass (kg), fat mass (kg), and body fat percentage (%)^15^. All measurements were performed by a trained nurse using a TANITA BC-418 MA instrument. Participants were instructed not to do anything special on the day of measurements and to resume their normal daily activities.

### Statistical analysis

Categorical variables were expressed as percentages (%) while numerical variables were presented as mean ± standard deviation (SD). Normal distribution of numerical variables was assessed by histograms. The participants were divided into four categories: WP smokers, cigarette smokers, dual smokers, and never smokers. Characteristics of the groups were compared using Pearson’s chi-squared test for categorical variables and one-way ANOVA followed by Bonferroni post hoc test for numerical variables. Bonferroni post hoc test was used to compare the difference in anthropometric measurements among the four groups. Similar analyses were used to explore the sex-stratified effect of smoking. Moreover, BMI, fat mass, lean mass and body fat percentage were included as the dependent variable in a multivariate regression analysis to test the relationship with the different types of smoking while adjusting for other confounding factors. Variables that were classified as confounders were age, sex, history of diabetes, and hypertension, as well as nationality which was considered a surrogate for diet, and genetic factors. Physical activity was similar in all groups and had no effect size on the analysis, hence, was not included in the final analysis. Results of the multivariate regression is presented as beta coefficients (β) and associated standard errors. All tests were two-tailed, and p values of less than 0.05 were considered statistically significant. Analyses were executed using STATA software version 16 (College Station, TX, USA).

## RESULTS

**Figure 1:**
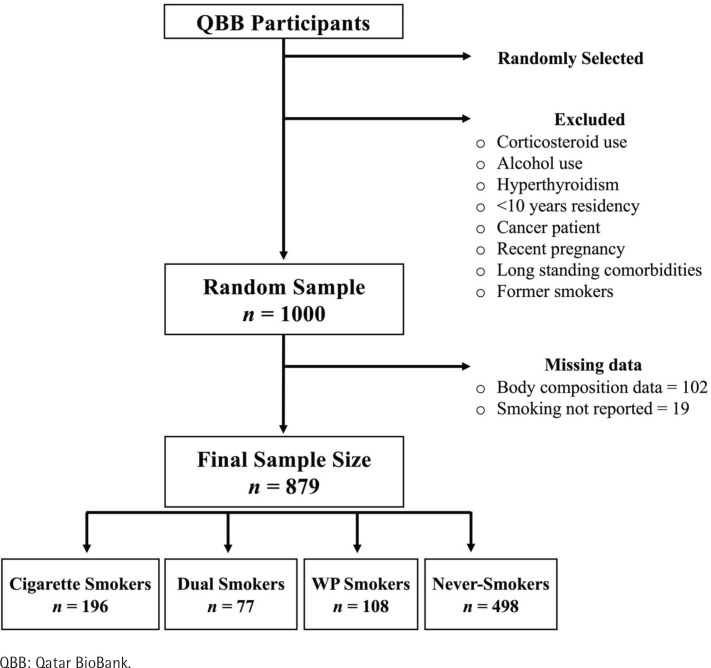
Flowchart depicting the size of the samples of the study and the smoking status of the study participants

A total of 1000 participants of the QBB were selected and, of these, 121 were excluded due to missing data on smoking status (19) and body composition (102) (Figure 1). The final sample of 879 participants was divided based on their smoking status into four groups: never smokers (498), WP smokers (108), cigarette smokers (196), and dual (WP and cigarette) smokers (77), as shown in Figure 1.

The dual smokers group consumed less cigarettes in comparison to exclusive cigarette smokers with a mean of 14 ± 9 cigarettes for the dual smoking group and 21 ± 13 cigarettes for the exclusive cigarette smokers’ group (p<0.001). Moreover, we noted that cigarette smoking is almost twice as prevalent as WP smoking amongst the participants.

The baseline characteristics of the sample group are illustrated in Table 1. The most common age group of the sample was 25–45 years. The average age of 35 ± 11 years was similar among the smoking groups except for a slightly older sample in the dual smoking group (38 ± 11 years). There was a statistically significant difference in gender among the four groups as WP smokers presented the only group with a female predominance (57.4% were females; p<0.001). While males comprised the majority (82.1%) in the cigarette smokers. Additionally, 82.6% of the participants in the study were Qataris, which were the majority in the four groups. There was no statistically significant difference among the four groups in physical activity in general and at work, with an equal proportion for both hypertension and diabetes in the whole sample (about 40%).

**Table 1:** Baseline characteristics of participants based on their smoking status

*Characteristics*	*Overall %*	*Never smoker %*	*Waterpipe %*	*Cigarettes %*	*Dual %*	*p*
**Total**, n	879	498	108	196	77	
**Age** (years), mean ± SD	35 ± 11	35 ± 11	35 ± 11	35 ± 11	38 ± 11	0.085
Male gender	62.7	58.8	42.6	82.1	66.2	<0.001
Qatari nationality	82.6	86.3	75.0	77.0	83.1	0.004
Hypertension	39.7	38.4	43.2	41.3	39.0	<0.001
Diabetes	39.3	33.7	40.7	51.0	42.9	<0.001
**Physical activity** (days), mean ± SD						
Moderate	0.87 ± 1.5	0.91 ± 1.7	1.01 ± 1.9	0.64 ± 1.5	0.96 ± 2.0	0.201
Heavy	0.67 ± 1.7	0.73 ± 1.6	0.56 ± 1.4	0.51 ± 1.4	0.83 ± 1.8	0.233
**Physical activity at work**						0.174
Sitting mostly	39.82	36.75	46.30	43.37	42.56	
Standing mostly	1.71	2.21	0	2.04	0	
Walking mostly	4.55	4.42	3.70	5.10	5.19	
Sitting, standing, walking equally	39.82	41.97	37.04	37.24	36.36	
Moderate physical work	8.19	10.04	6.48	4.59	7.79	
Heavy physical work	1.25	0.40	1.85	2.04	3.90	
Prefer not to answer	4.66	4.22	4.63	5.61	5.19	

Table 2 gives the BMI and body composition of the samples based on their smoking status. The average height was 167.38 ± 9.3 cm and average weight was 74.6 ± 17.7 kg, with height and weight significantly different between the four groups (p<0.001). Unadjusted analysis showed that the BMI of smokers, regardless of the smoking method, was significantly higher compared to never smokers (p<0.001). Moreover, 83.3% of WP smokers, 76.5% of cigarette smokers and 75.4% of dual smokers were either overweight or obese compared to 49% in the never smokers group. Furthermore, WP and cigarette smoker groups had higher fat mass (32.9 ± 12.3 and 29.0 ± 11.3 kg, respectively) than never smokers (23.4 ± 6.6 kg, p<0.001). *Post hoc* analysis showed that WP smokers had significantly higher BMI and fat mass when compared with cigarette smokers (p<0.05). Lean mass was also higher among smokers with a mean of 52.7 kg compared with 39.9 kg among never smokers. Conversely, body fat percent of WP smokers (36.1%) was similar to never smokers (35.7%) and higher than cigarette smokers (33.5%). Association between smoking status and sex-stratified anthropometrics are shown in the Supplementary file. There was a significant difference between the smoking groups and never smokers in the BMI and all body composition measurements in both males and females, except for the body fat percent where no difference was detected among the females.

**Table 2:** Anthropometric measurements of the participants based on their smoking status

	*Overall*	*Never smoker*	*Waterpipe*	*Cigarettes*	*Dual*	*p*
**Total**, n	879	498	108	196	77	
Height (cm), mean ± SD	167.4 ± 9.3	163.8 ± 9.1	171.9 ± 8.0	171.5 ± 6.8	173.6 ± 7.3	<0.001
Weight (kg), mean ± SD	74.6 ± 17.7	65.9 ± 12.2	89.2 ± 18.0	84.1 ± 17.0	86.4 ± 16.3	<0.001
**BMI categories** (%)						<0.001
Underweight	4.2	6.6	0	1.5	1.3	
Normal	34.1	44.4	16.7	21.9	23.4	
Overweight	44.6	49.0	38.9	39.8	36.4	
Obese	17.1	0	44.4	36.7	39.0	
Lean mass (kg), mean ± SD	45.5 ± 10.9	39.9 ± 9.4	53.2 ± 9.0	52.2 ± 7.8	53.4 ± 8.1	<0.001
Fat mass (kg), mean ± SD	26.39 ± 9.7	23.4 ± 6.6	32.9 ± 12.3	29.0 ± 11.3	29.9 ± 11.0	<0.001
Body fat (%), mean ± SD	35.1 ± 8.0	35.7 ± 8.1	36.1 ± 7.8	33.5 ± 7.6	33.8 ± 8.1	0.002

BMI: body mass index.

Table 3 illustrates the effect of smoking on BMI and body composition parameters in a multivariate regression model adjusted for age, sex, diabetes, hypertension, and nationality. The factors included in the model had a significant positive correlation with BMI (R^2^=0.24; p<0.001), lean mass (R^2^=0.35; p<0.001), and fat mass (R^2^=0.16; p<0.001); however, only a minor contribution was attributed to body-fat percentage (R^2^=0.05; p<0.001). Smoking, regardless of method, had statistically significant effect on BMI, lean mass, and fat mass after adjusting for other confounding factors (p<0.001). The effect was strongest on lean mass with a similar impact of both WP and cigarette smoking (β=12.9, SE=0.95; and β=12.2, SE=0.78; respectively). In addition, compared to cigarette smoking, WP had significantly stronger effect on BMI (β=3.8, SE=0.38; and β=5.5, SE=0.46; respectively), and fat mass (β=5.1, SE=0.79; and β=9.0, SE=0.97; respectively). In contrast, WP smoking had no effect on body-fat percentage (β=0.3, SE=0.84) while cigarette smoking and dual smoking were negatively correlated (β= -2.6, SE=0.69; and β= -2.1, SE=0.97; respectively) with body-fat percentage. This can be attributed to the greater increase in overall weight and lean mass in comparison to fat mass. The other factor that affected the weight and body composition parameters was history of diabetes which had a positive effect on BMI, fat mass, and fat percentage (p<0.001), but not on lean mass.

**Table 3:** Multivariate regression analysis^a^ showing the effect of smoking on anthropometric measurements of the participants (N=879)

*Variable*	*BMI*	*Lean mass*	*Fat mass*	*Body fat*
Waterpipe	5.5 (0.46)**	12.9 (0.95)**	9.0 (0.97)**	0.3 (0.84)
Cigarettes	3.8 (0.38)**	12.2 (0.78)**	5.1 (0.79)**	-2.6 (0.69)**
Dual	4.0 (0.53)**	13.3 (1.09)**	6.1 (1.11)**	-2.1 (0.97)*
R^2^	0.24**	0.35**	0.16**	0.05**

a In addition to smoking status, the model included age, sex, diabetes, hypertension, and nationality as independent variables. Data are presented as beta coefficient (standard error). BMI: body mass index.

* p<0.05;

** p<0.001.

## DISCUSSION

To our knowledge, this is the first study in the GR analyzing the association of WP smoking with certain parameters of obesity including BMI, fat mass, and body fat. In this population-based study of Qatari adults, daily WP use was associated with greater BMI and obesity. BMI of daily WP smokers averaged 5.6 units greater than never smokers. In addition, 44.4% of WP smokers were classified as obese compared to none in the never smoker group. Moreover, compared to never smokers, WP had significantly stronger effect on body fat mass. Although many of the WP smokers also smoked cigarettes, cigarette smoking did not significantly impact the WP/body weight association. This is in view of the fact that 44.4% of WP smokers were obese compared to 36.7% of cigarette smokers. Our results demonstrate a positive correlation between WP smoking and obesity, which is concordant with data obtained from studies in several countries including in the Middle East. Studies performed in Syria, Iran, Jordan, UAE, and Palestine showed that WP smokers, in comparison to non-users, are prone to developing abdominal obesity, BMI, metabolic syndrome, and diabetes mellitus^12^. Moreover, mice exposed to WP smoke had increased body weight, larger abdominal circumference, elevated blood pressure, and higher fasting glucose in comparison to unexposed animals^10^, plausibly due to metabolic dysfunction induced by tobacco smoke exposure. One of the plausible reasons for metabolic dysfunction can be the presence of gut bacteria. Studies have demonstrated an association of gut microbiota with human metabolic diseases and indicated susceptibility to obesity^16,17^. In addition, research has reported an association between smoking and human gut bacteria^18^. Lee et al.^18^ reported that human gut bacteria composition correlated with smoking. The study reported that in comparison to former and never smokers, current smokers had higher levels of bacteroidetes and the groups did not vary in terms of BMI or nutrient intake^18^. Nevertheless, the study also suggested cessation of smoking for a long duration can help the gut bacteria composition to recuperate to that of never smokers^18^. Additionally, both abdominal and overall obesity further contribute to cardiovascular diseases, diabetes, and metabolic syndrome^19^.

Although earlier reports demonstrated the harmful health effects of WP smoking, women, especially in the Middle East, tend to choose waterpipe over cigarettes^20^. Interestingly, our study revealed that WP smokers are predominantly female (57.4% were females; p<0.001). While, a multi-national study conducted in Middle-Eastern schools (students aged 13–15 years) revealed that boys from Gulf countries (Bahrain, Kuwait, Oman, UAE, and Yemen) except Qatar, are significantly more likely than girls to consume waterpipe^21^. Moreover, the study also reported that girls were more likely to smoke WP in comparison to cigarettes in all six countries (Bahrain, Kuwait, Oman, Qatar, UAE, and Yemen) analyzed^21^. Similarly, in a study by Memon et al.^22^, women in Kuwait represent 69% of waterpipe smokers compared to men (57%). Moreover, in Iran, intake of WP smoking is increasing quickly and becoming the most prevalent social and leisure activity amongst women^20^. In this regard, an increase by 75% in WP smoking was reported in women compared to men (14%)^23^. In the Kingdom of Saudi Arabia as well, there is a noticeable increase in WP use among women of different age groups; with school girls representing 14%, female university students representing 11%, while 16% are female doctors^24^. Moreover, studies in the Middle East (Lebanon, Iran, Egypt, Jordan, Occupied Palestinian Territories, and the United Arab Emirates) have shown that women smoke WP as frequently as men. A Lebanese study showed that 6.7% of women compared to 6.9% of men smoke WP, while the study reported a lower prevalence of WP smoking in women, however, higher waterpipe dependence was recorded in women (about 52%) compared to men (about 40%)^25^.

The Qatari population shows high rates of obesity in addition to other cardiovascular risk factors such as cigarette smoking, physical inactivity, diabetes, hypertension, and unhealthy diet patterns^26-29^. Furthermore, higher BMI and obesity is associated with the onset and development of several cancers including breast, lung, bladder, colorectal and prostate^30^; these cancers are prominent in several Middle-Eastern countries including Qatar^31^. Thus, it is evident that WPS can have an important role in the development of these cancers directly or indirectly.

Nevertheless, it must be mentioned that few investigations have reported lower BMI in cigarette smokers compared to never smokers^32^. This could be due to nicotine-mediating metabolic and anorectic effects^33^. On the other hand, results of other studies are in concordance with our findings with regard to the correlation between smoking and BMI^34^. This association is probably due to the collective obesogenic behavior including lack of physical activity, which may result in metabolic and anorectic effects of smoking^34^. Similarly, underlying mechanisms for the association of WP smoking and obesity can include lack of physical activity during the typically long WP smoking sessions, as well as the concept of socialization practiced around food and/or coffee in cafes and restaurants^35^. In our study, the plurality of WP users (46%) showed physical inactivity. Furthermore, reduced physical activity of WP smokers combined with lower nicotine intake, in comparison with cigarette smokers, may attribute to lower metabolic function in this group.

Interestingly, our sample showed that cigarette smoking is almost twice as prevalent as WP smoking, which is consistent with a recent study on the Qatari population showing that 21% of tobacco users consumed WP, while 43% smoked cigarettes^36^. Nevertheless, the recent trend of tobacco use decline presents a shift towards WP consumption, particularly from occasional or social use to daily and/or individual use^36^.

### Strengths and limitations

Finally, this general assessment of the association between tobacco consumption and obesity comes with certain strengths and limitations. One of the major strengths of the present analysis includes the random recruitment of participants from the QBB to avoid any potential selection bias, as the samples were not of those referred for metabolic syndrome. In addition, the use of Bonferroni analysis of variance enabled us to make anthropometric comparisons between the four groups to have a wider view of the results. However, our study has some limitations. Firstly, due to the exclusion and inclusion criteria used in the present analysis, the resulting cohort is not entirely representative of the general population in Qatar. Secondly, since data collection was done using a self-answered questionnaire, some of the participants might not have been comfortable with reporting their true smoking status, given the social stigma of smoking and the conservative nature of the Qatari society. Thirdly, the study lacked power to determine if associations between WP use and BMI were equally robust for both genders. Additionally, we were unable to determine the association between WP smoking and BMI in a dose-dependent manner. This was due to the fact that all WP smokers reported smoking only one WP session daily and did not report the number of refills. Lastly, the cross-sectional design of the study makes it difficult to establish a causal relationship between WP smoking and obesity. To further confirm the association, studies using larger prospective cohorts in diverse populations are required. In addition, prospective studies are required to elucidate the short-term and long-term effects of smoking cessation on obesity.

## CONCLUSIONS

This study demonstrates an association between WP smoking and BMI as well as fat mass. Daily WP users, compared to non-users, have a higher BMI and are more likely to be obese. Increase in body weight can elevate the potential risk of developing several health conditions including metabolic syndrome, diabetes, and coronary heart disease.

## Data Availability

The data supporting this research can be found in the Supplementary file.
