# Supplementary material for: Association between waterpipe smoking and obesity: Population-based study in Qatar
Source: Tob Induc Dis. 2022 Jan 26;20:06. doi: 10.18332/tid/143878 (PMC8788307; doi:10.18332/tid/143878)
Supplement: Supplementary file 1 [file TID-20-06-s1.pdf]

## Supplementary Material

**Supplementary Table 1.** Baseline characteristics of male participants based on their smoking status. Data are presented as mean ± standard deviation or as a percentage (%).

| | **Overall n=551** | **Non-smoker n=293** | **Waterpipe n=46** | **Cigarettes n=161** | **Dual n=51** | **P value** |
|---|---|---|---|---|---|---|
| Height (m) | 167.3 ± 9.3 | 163.6 ± 9.2 | 171.2 ± 8.6 | 171.0 ± 6.8 | 173.9 ± 8.0 | <0.001 |
| Weight (kg) | 74.7 ± 17.2 | 66.3 ± 12.0 | 86.5 ± 18.6 | 83.1 ± 17.2 | 86.3 ± 16.0 | <0.001 |
| BMI (kg/m2) | 26.51 ± 4.8 | 24.7 ± 3.4 | 29.4 ± 5.2 | 28.4 ± 5.5 | 28.5 ± 4.8 | <0.001 |
| BMI Categories (%) | | | | | | <0.001 |
| • Underweight | 4.0% | 6.1% | 0% | 1.9% | 2.0% | |
| • Normal | 32.8% | 43.3% | 15.2% | 23.6% | 17.6% | |
| • Overweight | 45.9% | 50.5% | 47.8% | 37.9% | 43.1% | |
| • Obese | 17.2% | 0% | 37.0% | 36.6% | 37.3% | |
| Lean mass (kg) | 45.7 ± 10.8 | 40.0 ± 9.4 | 52.8 ± 9.9 | 51.7 ± 7.7 | 53.5 ± 8.0 | <0.001 |
| Fat mass (kg) | 26.3 ± 9.5 | 23.8 ± 6.6 | 30.7 ± 11.7 | 28.4 ± 11.3 | 29.7 ± 10.5 | <0.001 |
| Body fat (%) | 34.9% ± 8.0 | 36.0% ± 8.1 | 34.9% ± 7.5 | 33.2% ± 7.7 | 33.6% ± 7.8 | 0.003 |

**Supplementary Table 2.** Baseline characteristics of female participants based on their smoking status. Data are presented as mean ± standard deviation or as a percentage (%).

| | **Overall n=328** | **Non-smoker n=205** | **Waterpipe n=62** | **Cigarettes n=35** | **Dual n=26** | **P value** |
|---|---|---|---|---|---|---|
| Height (m) | 167.4 ± 9.3 | 164.1 ± 9.0 | 172.4 ± 7.5 | 173.9 ± 6.4 | 173.3 ± 5.9 | <0.001 |
| Weight (kg) | 74.4 ± 18.5 | 65.3 ± 12.5 | 91.1 ± 17.4 | 88.9 ± 15.6 | 86.5 ± 17.2 | <0.001 |
| BMI (kg/m2) | 26.3 ± 5.0 | 24.1 ± 3.4 | 30.7 ± 5.6 | 29.3 ± 4.6 | 28.7 ± 5.0 | <0.001 |
| BMI Categories (%) | | | | | | <0.001 |
| • Underweight | 4.6% | 7.3% | 0% | 0% | 0% | |
| • Normal | 36.3% | 45.9% | 17.7% | 14.3% | 34.6% | |
| • Overweight | 42.4% | 46.8% | 32.3% | 48.6% | 23.1% | |
| • Obese | 16.8% | 0% | 50.0% | 37.1% | 42.3% | |
| Lean mass (kg) | 45.0 ± 11.2 | 39.9 ± 9.5 | 53.5 ± 8.2 | 54.4 ± 7.7 | 53.2 ± 8.4 | <0.001 |
| Fat mass (kg) | 26.6 ± 10.2 | 22.9 ± 6.6 | 34.5 ± 12.5 | 31.3 ± 11.1 | 30.2 ± 12.2 | <0.001 |
| Body fat (%) | 35.4% ± 8.0 | 35.2% ± 8.1 | 37.0% ± 7.9 | 34.7% ± 7.2 | 34.0% ± 8.8 | 0.3 |

© 2021 Alkeilani A.A. et al.
